# Can we halt health workforce deterioration in failed states? Insights from Guinea-Bissau on the nature, persistence and evolution of its HRH crisis

**DOI:** 10.1186/s12960-017-0189-0

**Published:** 2017-02-07

**Authors:** Giuliano Russo, Enrico Pavignani, Catia Sá Guerreiro, Clotilde Neves

**Affiliations:** 1 International Health and Biostatistics Unit, Instituto de Higiene e Medicina Tropical, Universidade Nova de Lisboa, Rua da Junqueira 100, Lisbon, Portugal; 2 Centre for Primary Care and Public Health, Queen Mary University of London, 58 Turner street, London, E12AB United Kingdom; 3 University of Queensland, Brisbane, Australia; 4 National Health Inspectorate, Ministry of Public Health, recinto 3 de Agosto, Bissau, Guinea-Bissau

**Keywords:** Human resources for health, Health systems under stress, Guinea-Bissau health system, Commoditised health markets, Health care in undergoverned countries, Health workforce crisis

## Abstract

**Background:**

Guinea-Bissau is one of the world’s poorest and least developed countries. Amid poverty, political turmoil and state withdrawal, its health workforce (HW) has been swamped for the last four decades in a deepening crisis of under-resourcing, poor performance and laissez-faire.

**Methods:**

The present study aimed at analysing the human resources for health (HRH) situation in Guinea-Bissau in light of the recent literature on distressed health systems, with the objective of contributing to understanding the ways health workers react to protracted turmoil, the resulting distortions and the counter-measures that might be considered. Through document analysis, focus group discussions, 14 semi-structured and 5 in-depth interviews, we explored patterns as they became visible on the ground.

**Results:**

Since independence, Guinea-Bissau experienced political events that have reflected on the healthcare arena and on the evolution of its health workforce, such as different coup attempts, waves of diaspora and shifting external assistance. The chronic scarcity of funds and a ‘stable political instability’ have lead to the commercialisation of public health services and to flawed mechanisms for training and deploying health personnel. In absence of any form of governance, health workers have come to own and run the health system. We show that the HRH crisis in Guinea-Bissau can only be understood by looking at its historical evolution and at the wider socio-economic context. There are no quick fixes for the deterioration of HRH in undergoverned states; however, the recognition of the ingrained distortions and an understanding of the forces determining the behaviour of key actors are essential premises for the identification of solutions.

**Conclusions:**

Guinea-Bissau’s case study suggests that any policy that does not factor in the limited clout of health authorities over a effectively privatised healthcare arena is doomed from the start. Improving health system governance and quality of training should take precedence over expanding HRH. A bloated and ineffective workforce must be managed through incentives rather than administrative orders, in order to improve skills and productivity against higher remuneration and better working conditions. Donor support might be crucial to trigger positive changes, through realistic and sustained investments.

## Introduction

Human resources for health (HRH) has long been recognised as a critical component of any health system [1], due to their large financial implications [2] and the way they shape its functioning. In situations of conflict and prolonged governance disarray, health systems get critically distorted, with consequences lasting well into the years after the end of turbulence [3]. The health workforce gets particularly affected by violence, as its numbers in the field decrease, professionals are victims of attacks and migrate, their skills decline, while accelerated and unplanned training may expand the supply of underqualified health personnel [4].

Attempts have been made in the past to conceptualise post-war health recovery and human resources for health [5], with the objective of identifying the support needed to overcome the flaws induced by protracted stress [6]. A review of the HR field [7] concluded that while aspects such as supply, education and recruitment have been investigated in post-conflict situations, others such as deployment and governance have not been adequately covered. Some have explored political economy aspects of human resources for health and of related policies [8–10], as well as the role of human resources for health in the state-building process following an armed conflict [11]. The complex remuneration of health workers, generated by multiple activities in undergoverned contexts, have also attracted the interest of scholars [12, 13].

Where the state is absent, underfinanced or plainly failed, the applicability of widely held concepts is called into question. The conventional view of a national health system covering a clearly demarcated territory managed by recognised health authorities does not apply to many healthcare spaces multiplying in the global South [3, 14]. Thus, standard health policy and planning tools fall short of capturing the complexities of distressed systems, not least because of the paucity of reliable health and systems data in such circumstances [4].

Guinea-Bissau offers itself to scrutiny as one of the poorest and most dysfunctional states in the world. Unlike other equally derelict countries, possibly due to its small size, marginal strategic value and lack of natural resources, Guinea-Bissau and its health system have received little attention from the press, the international community and academia [15]. The present study aimed at analysing the HRH situation in Guinea-Bissau in light of the recent literature on health systems in fragile states, with the objective of identifying the key forces shaping the development of its health workforce, the resulting distortions and the counter-measures to be considered.

## Background

Swamped in political instability, underdevelopment and stalled economic growth since its independence [16], Guinea-Bissau exemplifies the state that, far from having ‘failed’, has never really functioned as expected [17]. During the last 40 years, the country has experienced socialist rule, civil war and prolonged political and military unrest, until becoming a hub for international drug smuggling routes [18]. The latest military coup in connection with the 2012 presidential elections came to shake once again the precarious balance of power; after the 2014 elections, a recognised government is currently in place [19]. In 2014, the country was ranked 176th out of 187 in the UN Human Development Index [20, 21] with the poverty headcount ratio at 69.3% in 2010—up from 64.7% in 2002.

Guinea-Bissau has a small economy, with a gross domestic product (GDP) of just above 1 billion USD (550 USD-PPP per capita). Economic growth averaged 2.5% in the last 5 years, although following the resumption of donor financing after the latest *coup d’état* and thanks to sustained international prices for cashew nut—the country’s key export—the economy expanded by 5.1% in 2015 [22]. Net Overseas Development Assistance accounted for 10.6% of the gross national income and financed 46% of public spending. Dominated by non-tax sources, such as customs duties and fishing licenses, internal revenues represented 14% of the country’s wealth. In 2015, the wage bill accounted for 51% of the government budget [23].

Total health spending is estimated at 5.6% of GDP, with just 21% supported by public funds, and out-of-pocket expenditures representing 62% of private contributions [20]. The Ministry of Public Health (MoPH) was allocated 8% of government budget in 2015, with salaries taking up 72% of public health expenditures [23]. It is a shared opinion that Guinea-Bissau’s future fiscal position does not bode well for its poverty reduction plans.

Life expectancy in Guinea-Bissau is estimated to be 55.8 for women and 52.8 for men, with under 15-year-olds representing 42% of its population [21]. The country’s epidemiological profile is dominated by poverty-related communicable diseases, such as respiratory infections, malaria, AIDS (prevalence of 3.9%) and tuberculosis [24]. Successive multiple indicators surveys have shown a substantial reduction in infant mortality in the last decade, estimated in 2014 to be around 55/1000; 32% of under five children were found to be underweight. Some recent UN estimates put maternal mortality at 900/100 000 [24]. The country’s health system structure has changed little in the last 40 years, with a wide base of basic health units supported by community health workers and traditional birth attendants, health centres staffed by civil servants, primary health services organised around outreach programmes, five regional hospitals with some surgery capacity and one central hospital located in the capital city—the Simão Mendes Central Hospital [25, 26]. Overall health coverage was estimated to be 34% in 2010, down from its pre-war level of 40% [27].

Despite recent attempts to revitalise its health system, health indicators are sagging, while its already shaky health workforce is deteriorating further [28]. In 2015 almost 27 956 assisted deliveries and 171 135 ante-natal care visits were recorded for a population of 1.5 million (Table 1 below). Just 2221 Caesarean sections were performed, 66% of which in the capital city area; during the same year, just 1.4 new admissions were recorded per surgical bed, 5.1 for obstetric and 2.6 for general medicine ones.

**Table 1:** Public health system selected in-patient and outpatient activity indicators (2015, 12 months)

Province	Population	Mother and child care				In-patient surgical care		In-patient obstetric care		In-patient general medicine care	
		Assisted delivery	Caesarean sections	Complications-related transference	Antenatal visits	Admissions	Beds capacity for the specialty^a^	Admissions	Beds capacity for the specialty^a^	Admissions	Beds capacity for the specialty^a^
Bafatá	214 541	3257	228	267	25 217	103	156	2194	286	3941	1034
Bijagos	23 728	452	0	11	3315	1009	96	124	54	669	393
Biombo	99 364	2257	37	209	13 708	225	1	1792	99	2164	401
Bolama	10 900	169	0	4	942	0		26	108	291	487
Cacheu	197 634	3121	138	386	24 116	203	394	1024	569	2286	1055
Farim	51 545	847	4	139	7329	13		781	80	604	253
Gabú	219 586	3681	135	249	28 212	168	188	2322	277	906	135
Oio	178 348	2350	147	392	19 400	11	157	288	213	1206	862
Quinara	64 909	896	0	75	6554	45	61	712	345	2253	777
Greater Bissau (SAB)	389 918	9345	1467	451	29 707	1338	1130	9535	1582	10 177	3493
Tombali	97 282	1581	65	114	12 635	67	85	1190	296	1614	1059
Total	1 547 754	27 956	2221	2297	171 135	3182	2268	19 988	3909	26 111	9949

Source: INASA (2016): Boletins mensais de 2015

^a^Number of beds available are not cumulative, as these are used and separately counted for different specialties

## The methodology used

**Fig. 1:**
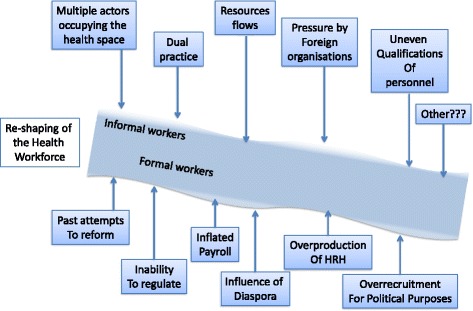
Conceptual framework to understand the evolution of Guinea-Bissau's health workforce through shaping factors and ensuing distortions

We started from the assumption that, because of its key exogenous and endogenous shaping factors, a health workforce acquires specific features—the distortions—which are essentially similar to what is observable in other distressed contexts, even if the mix and intensity of each are distinctive of the country under analysis (Fig. 1). Crucially, the boundaries between formal and informal workers active in such health workforce are often blurred, and it is hard to acquire a firm idea of its true size [29].

Through document analysis, focus group, semi-structured and in-depth interviews, we aimed at assessing the presence and combination in the Guinea-Bissau case study of the aspects observed in other troubled settings, such as (a) the empty void vs. crowded space characterisation [14], (b) the commercialisation of health services [30], (c) the influence of different forms of multiple employment [31], (d) resource flows into the system [10], (e) inconsistent engagements of foreign organisations in the health arena [32], (f) poor quality of training, (g) diverse qualifications of health personnel [33], (h) over-recruitment for political purposes, (i) imbalanced distribution of personnel, (l) failed past attempts to reform the sector [9], (m) inflated payrolls and (n) the influence of the diaspora on the labour market.

### Data collection and data analysis

Published and unpublished documents in Portuguese and English from the 1970s and 1980s were first reviewed on (a) the historical and political crisis in Guinea-Bissau, (b) the Guinea-Bissau health system and its evolution, (c) health policy documents and (c) health systems under stress. PubMed, Scopus, Googlescholar and EconLit databases were searched for terms such as ‘Human Resources for Health AND Crisis’, ‘Guinea Bissau AND Health’, ‘Conflict AND Human Resources’, ‘Portuguese-speaking African countries AND Human Resources for Health’, ‘Diaspora AND Human Resources for Health’, ‘Health systems AND Post-war Reconstruction’. The World Bank and WHO health databases and UNICEF MICS across several years were used as data sources.

Data on the characteristics and deployment of the current health workforce of Guinea-Bissau and on training outputs were obtained from the MoPH’s National Directorate for Human Resources (DNRH) and from the National School of Health (ENS). An original Excel database was built containing information on sex, age, category, current deployment and remuneration for each individual health worker currently employed in the health sector.

An inception focus group [34] was conducted with five health officials purposively selected by and among the country’s current ENS training institution in Bissau to brainstorm ideas on the evolution of the health workforce since independence, on the key distortions and shaping factors, and on potential informants for the interviews. Fourteen semi-structured [35] and 5 work and career history in-depth interviews [36, 37] were conducted in Lisbon, Bissau and Bolama districts by two researchers. Key informants were selected among policy-makers, government officials, health workers and international health organisations personnel and identified through a snowball technique. Interviews were stopped when saturation point was reached for the key themes.

The semi-structured interviews touched on (1) the interviewee’s appraisal of the current HRH situation in Guinea-Bissau, (2) his/her opinion/knowledge on its evolution, (3) identification of key turning points that modified the HRH along the last decades, (4) personal perceptions of key problems and (5) probing the key distortions identified from the literature (see the Interview Guide in Appendix 1). Work history interviews explored health workers’ experience of entering the health system, his/her recruitment, training, deployment, current and past work routines and sources of revenues (see Work History Guide in Appendix 2).

Interviews were conducted in Portuguese between February and March 2016. Semi-structured interview lasted for between 45 and 90 min. In-depth life story interviews lasted for over 2 h. They were all audio-recorded and analysed for contents, according to the shaping factors and distortion categories identified in the literature; then, they were traced and triangulated across interview groups; finally, a narrative of events was constructed for the evolution of the crisis, stakeholder influences, dominant distortions and their combination. Individual informants were asked to confirm specific narratives during the paper’s writing-up to ensure internal validity.

## Results

### Evolution of the health workforce

**Fig. 2:**
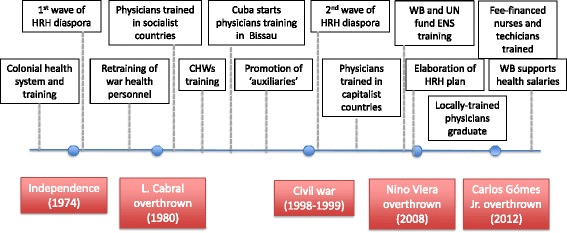
Timelines of historical events and their repercussions on Guinea-Bissau’s health workforce

As with other African countries, in colonial Guinea-Bissau, health services were essentially curative and hospital-based, designed around the needs of the white population residing in or visiting the country [38]. Physicians and other senior cadres were either from Portugal and Cape Verde, while lower-level health workers were trained locally in the main healthcare institutions. With independence from Portugal in 1974, the totality of foreign physicians and a considerable proportion of older Guinean nurses fled the country, attracted by the prospect of claiming retirement benefits from the new Portuguese State, in what was identified by the interviewees as the first wave of health workers’ diaspora (Fig. 2).

Re-training and integration of war health auxiliaries—known as *socorristas*—started in 1976 in the Nhala and successively in the Bolama training schools. Because of the connections created during the liberation struggle, the then-USSR and Cuba offered to Guinea-Bissau medical training scholarships, with the objective of filling the void left in the health system by the decolonisation process. The 1980 coup overthrowing first president Luís Cabral was seen as a sea change for Guinea-Bissau towards a market-oriented economic model; for many interviewees, this shift impacted on the motivation of health workers to serve in the public sector (see the ‘Commercialisation of public services’ section).

Community health workers (CHWs) were trained in the 1980s by European non-governmental organisations (NGOs) [39], and their utilisation was consolidated in connection to the launch of the Bamako Initiative in the country, a revolving drug fund scheme aimed at financing healthcare provision [40]. The National Health School (ENS) was established between 1992 and 1995 in Bissau, with the objective of training and upgrading nurses and health technicians, while the local university started training physicians with Cuban support. In this short period, the World Bank and UN agencies funded HWs’ training (Fig. 2).

A first attempt to define a HRH plan was carried out in 1996, with the aim of consolidating the gains of a primary care-oriented strategy previously implemented [41]. However, the 1998–1999 civil war buried the feeble progress made in health workforce development in the previous 25 years. Amid security concerns, those who could fled the country, in what was identified by our interviewees as the 2nd HRH diaspora wave. Recently trained senior cadres left, aided by a relaxation of hosting requirements set by the international community for refugees.

[…] in the first diaspora it was mostly elder nurses who left, but now it was physicians, managers and senior government officials…. We were left with nobody to run the [health] system. Policy-maker-003.

The practice of referring patients for care abroad—with a health worker accompanying his/her ailing patient—became common, representing a corridor for physicians desperate to leave their beleaguered country. Training abroad, however, did not stop during the war years, and many of the senior doctors these days in key government positions received their education in that period, mostly in European universities.

After successive coups and election rounds, João Bernardo (Nino) Vieira established himself as the country’s strongman in 2004; this event brought back funds and international support for the health sector. The Cuban Brigade returned to train physicians in Bissau in 2005, with the World Bank funding the restructuring of ENS, its mid-level training courses, and the development of the second National Plan for Human Resources Development (PNDRH II) through 2008 [42]. When Nino Vieira was assassinated, the country was hurled back into political turmoil, with the international community focussing more on peace building than on health service provision [19].

The first locally trained physicians started graduating and entering the health system in 2013, and the ENS began to run nursing and technical courses funded exclusively through student fees. The economy started to grow again, in particular in the capital city Bissau Autonomous Area, today home to a third of the country’s population [20].

### Key forces shaping the development of the Health Workforce in Guinea-Bissau

Scarcity of funds and political instability emerged as the two key forces shaping human resources for health in Guinea-Bissau between independence and modern days. In absolute terms, markedly little money is currently allocated to health salaries by the state budget; the total wage bill for the 2173 employees in the health sector in 2015 was XOF216 million per month (US$393 392). Remarkably, the State only pays for healthcare-related salaries, with all other expenses (medicines, goods and services, investments) being partially covered by external funds and by health facilities’ own revenues. Incomplete data on external assistance combine with absent information on the user fees paid to obscure true financing levels.

Together with education workers, public jobs in the health sector are widely considered privileged in comparison to other civil servants, having been protected against cuts. Health employees’ average salary was US$169 per month in 2015, with nurses paid on average US$177, physicians US$291 and specialists US$380 [43]. Salaries are typically paid with considerable delays—although with arrears—and it is considered the norm for new recruits to receive their first payments only 1 year after starting the post. Failure to pay regular salaries to health workers was mentioned as one of the causes of recent strikes. After the latest contested election results, the World Bank agreed in 2014 to provide earmarked funds for health personnel expenditures from its Social Protection programme, in a move intended to avoid health workers walk-outs, decrease the rising tension and minimise the risk of civil unrest. The state’s inability to provide for its employees was seen by many as one of the key weaknesses of the system.

If you cannot even pay for salaries, you’re not a State; you are just an added coach to the World Bank’s train! Government official-001

Health and education payrolls were cleaned and consolidated between 2014 and 2015, and a new personal bank account-based payment system introduced to eliminate ghost workers in these sectors, the two largest public employers. However, the World Bank’s support to personnel expenditures was terminated for alleged management irregularities and for the government’s failure to avoid health workers’ walk-outs in December 2015.

The term ‘political instability’ (PI) was called upon by all our interviewees as the major determinant of the country’s current situation, with pervasive ramifications for the health workforce. Since independence, the country has experienced 18 coup attempts, and recently, three health ministers were changed in as many years [28]. HRH development is forcibly undermined by such constraint. Additionally, domestic turmoil provokes donor reactions, in turn affecting the resources allocated to healthcare provision. On the other hand, the ritual invocation of PI offers a convenient screen behind which health workers adopt questionable behaviours, and managers abstain from sanctioning them.

The term ‘political instability’ was used loosely by our informants, at times employed to refer to the phenomenon of governance failure, and other times to describe its consequences. At least five different interpretations of this concept were identified, namely:

- Political instability as the recurring turnover of politicians, policy-makers and mid-level managers, which would prevent policies being upheld, and plans implemented. Therefore, such officials could not be held responsible for carrying out demanding tasks and for taking initiatives. The awareness of the limited time they are likely to spend in office breeds short-termism among government officials, who therefore dedicate their efforts to seize low-hanging fruits in the best of case, or to get access to public resources in the worst.
- PI as disruption of financing flows, as after each coup attempt or civil unrest public sector salaries and foreign-sponsored health programmes get interrupted—this was described by one informant as ‘turning off the tap when the situation cyclically deteriorates’.
- PI as an inherent vulnerability of all public institutions, exposed by the slightest of adverse events, coupled by an incapacity to put up a response at any level—a sort of systemic vulnerability (‘[…..] our country’s like a patient without his immune system: any issue turns into a disease’. Health worker-005.
- PI as lack of economic development stemming from the country’s weakness. At times used as an excuse for anything wrong in Guinea-Bissau, some of our informants explained that ‘….patients don’t have the money to pay for [user] fees because of the political instability’. Health worker-002. Poverty, and the state frailty induced by meagre tax yealds, is therefore conflated with turbulence, a manifestation of, as well as an aggravating factor for state withdrawal.
- PI as inability of the government to exert power by controlling, monitoring and evaluating the application of the rule of law and frequently being itself an egregious violator of it.

International aid in the health field has changed considerably in direction and intensity over the years, first inspired by geopolitical motives in the years following independence, and more recently motivated to a large extent by global security and drug smuggling control concerns [19]. The development of the national health workforce has been swayed by the Portuguese former colonial power offering refuge to professionals during the two diaspora waves, as well as by the ideologies and technical expertise of those former communist bloc countries—particularly Cuba—offering opportunities for training abroad first, and then creating capacity for training physicians locally. Far from remaining a domestic process, HRH development has been affected by multiple international forces. This crucial aspect, regularly missed by traditional policy and planning approaches, is becoming the norm in an increasingly globalised world, particularly in small countries with open borders [44].

Lower-level training has been intermittently supported by UN agencies and the World Bank, as well as by a short-lived attempt in the 1980s by the Dutch cooperation to train Community Health Workers [39]. Because of the comparatively limited interest and involvement of bilateral international agencies with successive governments, non-government organisations have traditionally played a substantial role in health service delivery in Guinea-Bissau. Faith-based organisations—particularly those linked to the Catholic Church—were among the few to remain in the country during the war years; they are still considered one of the few providers of quality services in the country, particularly those in the capital city area.

More recently, the international aid community has withdrawn from funding HRH training; it is however striking how donors have adopted diverse positions in the Guinea-Bissau’s health arena, ranging from the European Commission’s decision to unilaterally withdraw from engaging public health institutions, to the World Bank earmarking of salary funds for the social sectors. NGOs—particularly Portugal-based ones—have since thrived, ready to occupy the space and funding previously channelled to activities carried out by the national government; the large EU-funded Integrated Mother and Child Health Programme is largely managed though NGOs contracts.

For Guinea-Bissau, aid dependency translates into accepting donor agendas, with their proliferating priorities, changing fashions and de-contextualised decision-making. Some of these agendas have heavily shaped domestic developments and not always in the way expected by aid agencies. The Bamako Initiative is illustrative of such pattern; its impact on the health workforce, and in turn on access to health care and on its quality, cannot be ignored.

### Commercialisation of public services

Together, the forces described in the previous section have commoditised healthcare provision, as witnessed in healthcare arenas as diverse as Cambodia [45], Lebanon [46] and Somalia [47]. It is an open secret in Guinea-Bissau that fees are charged for any kind of health care. Statutory fees—and respective exemptions—are defined for specific services and drugs, with the stated objective of recovering drug costs and providing health facilities with a management fund; but in practice, such fees have been hijacked by health professionals, with charges reported to be erratic, arbitrary and at times unreasonable. While this ubiquity of illegal charges was recognised for the comparatively prosperous capital city area [31], our interviews revealed the practice to be thriving also in the poorest rural areas.

[…] No sir, this is the way it works here [in Bolama]: treatment for uncomplicated malaria is worth XOF7,000 [$12.72], complicated malaria cases [involving intravenous drips and second line treatment] twice as much, and a Caesarean section in Simão Mendes Central Hospital is XOF37,000 ($63.3). Health worker-001.

As no financial system seems to be in place to truly recover the costs of the resources used by the facilities—apart for the Bamako Initiative-inspired drug revolving funds through which health care units purchase their drugs at subsidised prices from the central drug store—revenues from charges are entirely captured and managed by health workers acting as managers (‘No money goes back to Bissau, just the [health statistics] data’. Health worker-002)*.*

Unaffordable charges were identified as the true reason behind low service utilisation in the poorest rural regions. While the existence of a flourishing folk medicine—divided between medical plants healers (*djambakôs*), Islamic faith therapists (*mouros*) and shamans (*curandeiros*)—is documented for Guinea-Bissau [48], little evidence was found of informal health practitioners for the poorest rural areas. For the comparatively richer and more dynamic Bissau area, informal drug stores (*boutiques*) were reported as often recruiting health workers from the public service to offer a sort of ‘medicines cum informal consultation’ service.

This system of informal charges was touted to be so institutionalised among health professionals and users alike, to be taken for granted as the official way of supplementing meagre and irregular salaries in poor regions and to make a decent living in the more expensive capital city. The health workers interviewed declared seeing the issue of charges as intimately related to their low and erratic remuneration. Many declared feeling ‘abandoned’ by the State, which barely pays for salaries, but leaves health professionals fending for themselves for recurrent expenses. As a result, many stated having to turn into managers to use these unofficial resources to run public services, purchasing drugs, hiring local support staff and paying for transport and maintenance. Predictably, informants did not mention the portion of fees they pocket.

….with that money you have to first pay for drugs. Then you pay for petrol and small maintenance repairs. Then you pay for the ‘locally contracted’ staff. But as this month the money is tight, I have already told them they will have to wait for next month to be paid. Health worker-003.

As no effective inspection system is in place, the nature and extent of charges and mark-ups were reported to depend exclusively on the goodwill (and creativity) of the most senior officials in charge, on their ability to enforce those charges, and on the market to take the price.

I am not against the health service charges, but to me the real shame are the ‘fines’ some health workers charge to those women who do not deliver in the health centre, or do not bring their kids to get a jab. NGI-001.

Although complaining about meagre public earnings, as well as the distance from the capital city, health workers seemed to have adjusted to the current living conditions, allocating time to other daily chores and alternative profit-generating activities.

…I wake up very early every day. Before going to work I have to procure ‘mafé’ [a meal’s protein ingredient accompanying the staple rice dish] for my family’s dinner. Then I go to work. Now that it’s the cashew nut harvest time I have to leave early to check on the workers tending my trees. HW-002.

Commercialisation pervades the healthcare arena beyond the fees charged for the services provided. Professional training has been turned into a business, offering another manifestation of the same process, as described in the next section.

### Distortions in HRH training and deployment

The public health sector currently officially employs 2173 workers in Guinea-Bissau, of which 264 physicians and 1027 nurses. In relation to the served population, there were 1.7 physicians and 11.5 health workers per 10 000 inhabitants in 2016. Over the recent years, the impact of the war-related diaspora on the workforce has been noticeable, particularly in terms of the loss of skilled cadres between 1996 and 2007 (Table 2).

**Table 2:** Evolution of the health workforce between 1996 and 2016, by categories

Category	1996	2007	2016
Physicians	165	104	264
Nurses	357	300	1027
Midwives	67	177	141
Technical staff	276	199	244
Support	417	642	98
Other (aux.)	1043	696	399
Total	2325	2118	2173

Sources: PNDS I (1997), PNDS II (2008), DNRH (2016)

Although on balance, the health workforce has been relatively stable during the last two decades, progress has been registered in terms of the upgrade of auxiliary health personnel, and of the reduction of support staff in favour of training general nurses and physicians [41, 49]. While the overall number of physicians has grown by 37.5% in 20 years—enough to offset the significant decrease registered in the post-war years—the nurses employed in the public system experienced an almost threefold increase in the last decade.

Interestingly, despite the lack of resources and low service utilisation, additional support staff is often recruited locally, including non-health personnel, retired technical staff or recently qualified health workers waiting to be appointed. These health workers tend to escape reporting, both in relation to their presence and remuneration.

…yes, I worked here as a lab technician for over 40 years. When I retired, I offered to continue supporting the health centre with my expertise. But with the new [bank-based] payment system I stopped receiving my salary, and I receive payments irregularly, depending on the tasks I carry out. HW-004

In 2016, 60% of all health workers were female, although women represented only 31% of physicians. The nurse to physician ratio was 3.89 for the whole country, quite a balanced, although unplanned one. The ratio of combined nurse, auxiliary nurse and midwife to physician was 4.84, with substantial differences between the ratios in the capital city (3.05) and remote rural areas such as Quínara e Bijagós (16.00 and 14.67, respectively).

Although staff distribution is heavily influence by distribution of hospital beds, substantial geographical imbalances emerge, as 51% of all physicians, and 40% of all nurses are still based in the Bissau Autonomous area, home to just 25% of the country’s population. Populous regions such as Bafatá and Gabú show a systematic disadvantage in the deployment of all types of health personnel in favour of the Bissau and neighbouring Biombo areas (Table 3).

**Table 3:** The officially recorded Health Workforce in Guinea Bissau, by category and regional deployment (2016)

Category	S.A.B. Bissau		Other provinces		Total	
	No.	As % of total (%)	No.	As % of total (%)	No.	As % of grand total
Population	389 918	25	1 157 836	75	1 547 754	100
Specialised physicians	15	54	13	46	28	1
General practice physician	119	50	117	50	236	11
Other higher education personnel	128	80	33	20	161	7
Nurses	409	40	618	60	1027	47
Midwives	75	53	66	47	141	6
Technical staff	147	60	97	40	244	11
Auxiliary	39	74	14	26	53	2
Auxiliary nurses	50	45	61	55	111	5
Administrative personnel	58	78	16	22	74	3
Support staff	59	60	39	40	98	5
Grand Total	1099	51	1074	49	2173	100

Source: DNRH (2016). Locally-recruited cadres not included in this count

Arbitrary deployment was widely reported to be a key issue; although attempts have been made to establish a Deployment and Transfer Commission defining rules and standards to allocate new personnel to health regions according to needs, distributing health workers remains a largely opaque process, subject to political pressures and trading practices.

…we are trying to set standards, rules and procedures, but so far the commission has not met regularly, and if we receive a request from high-up to transfer somebody to Bissau, we have no way of saying no. Health official-002.

Staff deployment driven by the motivations of health workers rather than health service needs is a widespread phenomenon [50], only more visible in an undergoverned healthcare arena. Compounding the frailty of management structures, the inadequate enticements offered to staff reluctant to accept hardship posts cannot redress the strong forces at play. Moreover, health authorities short on relevant information could not conceivably decide about actual service needs.

Health personnel training increased dramatically between 2009 and 2015, with the reformed ENS graduating 1125 health cadres [51], against the few hundreds projected by the PNDRH, and the local Faculty of Medicine graduating in excess of 100 general physicians with the support of the Cuban Brigade. The training of nurses in particular has expanded, despite failing to receive any external or state fund—with 829 new nurses over 7 years. Since 2009, recurrent expenditures for such courses have been supported exclusively by student fees, in the Bissau main training school as well as in its southern outposts (*polos de formação*), these latter now discontinued. Despite the relatively high fees (approximately US$500 over the 2-year course), such courses were reported to be routinely oversubscribed.

For every nursing course we have 100 openings, but at times we receive over 2000 applications! […] - but the quality of such applicants is not good – sometimes we have to lower the minimum grade accepted, and go down to 9 grades [out of 20]. Policy-maker-005.

Physicians are both trained abroad and locally, through the Cuba-supported local Faculty of Medicine; more recently, two private medical schools started operating in Bissau, although programmes and facilities are still to receive accreditation from the MoPH, and one of the schools was shut down for irregularities in 2016.

The majority of the interviewees identified quality of training as a pressing concern. Although training curricula were reformed and updated, factors like (a) applicants’ education level, (b) poor competence of teachers, (c) high teachers to students ratios and (d) sub-optimal practice sites, were cited as key constrains.

The new nurses we get here sometimes can’t read and count properly, not to mention mastering the basics of clinical care. The old ones that should teach them have already gained too many bad habits, and are plainly not interested in passing on their expertise. NGI-001.

Local church and NGO representatives working with clinical personnel claimed to have needed to retrain the health workers assigned by the MoPH before employing them in their own facilities, for lack of the required skills.

No Ministry of Health (MoH) in-service training programme exists for updating and developing the skills of the national personnel, with the initiative left to NGOs and international agencies, launching specific training programmes as they see fit. At the MoH level, supervision is the responsibility of the underfunded Central Inspectorate Unit, consisting of one senior official and two support staff. Regional Health Directorates are also charged with conducting inspection visits to their health facilities, but no specific budget lines are made available for this purpose.

## Discussion

This study of Guinea-Bissau’s health workforce not only confirmed many of the attributes found elsewhere in undergoverned countries but also presented original features worth highlighting. Privatisation from within, commoditisation of health care, privately-sustained production of health workers, are some of the recognisable characteristics Guinea-Bissau shares with other countries displaying underfunded health systems in severe disarray [29]. Such developments occurred against an official backdrop of public health provision, with health professionals eager to enter the civil service, and health authorities keen to offer jobs and other related perks. However, the persistent scarcity of domestic funding and fluctuating external assistance have exposed such patterns in clear contours in Guinea-Bissau, setting it aside from other countries such as Angola, where oil revenues have allowed an unchecked expansion of the civil service, and Mozambique, whose health sector has been propelled by generous aid flows [33].

Political instability appears to be a key shaping force of Guinea-Bissau’s domestic environment, whether this term is used to describe the government’s inability to exert power, its fragility or the continuous disruption of resource flows. Rather than the exception, political instability has come to represent the norm for Guinea-Bissau: future attempts to revitalise the health sector will have to factor in this permanent turmoil, as the country has not really known any other form of operating since independence.

An extreme case of ungoverned health workforce is possibly what we witnessed in Guinea-Bissau. Left to their own devices, abandoned by their official employer*,* with little or no supervision, health workers have gradually become the real owners and operators of health services, run to their advantage and regardless of their worth to the users. Arguably, the Bamako Initiative has been appropriated as a convenient fig leaf behind which the commoditisation of healthcare provision has progressed, rather than providing a lifeline for cash-strapped health systems, as reportedly happened in other West-African countries [52, 53].

That health workers come to own an undergoverned health system has been recognised also elsewhere [54], although such crucial aspect is being inadequately considered in the policy and planning discourse. In Guinea-Bissau’s case, civil service jobs represent one of the few opportunities in the country’s tight formal labour market, which explains the willingness of prospective candidates to pay hefty fees for low-quality professional training. Health workers accept low and irregular salaries because these are probably the closest thing to a steady source of revenue. A public appointment is made more attractive by the freedom they enjoy to embark on an array of other profit-generating activities connected with their position.

As witnessed in other distressed contexts, such as the DR Congo [5], the health training system has grown spontaneously, fuelled by its own earning imperatives and market demand. As suggested for the DR Congo case, technical measures are unlikely to address the discussed shortcomings, nor will the health officials presiding over (and benefitting from) them, be the committed enforcers of risky and controversial structural reforms [13].

Counter-intuitively, Guinea-Bissau’s health workforce looks oversized - particularly once locally-contracted staff are considered—vis-à-vis its health service outputs and meagre domestic resources, present and forecast. The inevitable attraction of richer urban areas, rather than the absolute lack of health professionals, is the likely cause of HRH shortages in the poorer parts of the country. And such earning perspective motivates young people to acquire a healthcare qualification. The quality of the services provided by Guinea-Bissau’s health professionals is inadequately studied, but it is likely to be substandard. The PNDRH provided an argument for the expansion, rather than for the regulation of the workforce [55]; but expanding a derelict health workforce without addressing its current faults is likely to make them more severe and irremediable.

Can anything be done to stop the deterioration of the health workforce in undergoverned countries like Guinea-Bissau? The scarcity of reliable information is striking, and the fluidity of the situation compounds matters, curtailing the shelf life of the available data; the MoPH (and the state administration at large) lacks the analytical, financing, legal and managements levers needed to intervene successfully in a field that has evolved spontaneously and now responds to market signals more than to administrative instructions. To offset such shortcomings, external assistance should be strategic and sustained overtime, which is seldom the case for fragile states [32]*.*

What realistic steps should be taken to develop the health workforce in Guinea-Bissau, or at least to contain its deterioration? The recognition of the current picture is sorely needed—by national and international policy-makers alike; the image of a well-meaning, understaffed and underfunded public health system struggling to bring health services to the population needs debunking. Amid Guinea-Bissau’s power vacuum and lack of employment opportunities, health workers have come to own the public health system and provide erratic, low-quality and payment-only services to make a living. In Guinea-Bissau’s case, this is the ‘elephant in the room’ of its public provision of health care, and it is consistent to what observed elsewhere in similar circumstances [56, 57]; any assessment that does not confront such obvious although uncomfortable fact would generate misconceived reform attempts.

A rational approach to identify solutions in undergoverned states should start with the macroassessment over time of the resource envelope, which would usher in a discussion among stakeholders about what sort of health system could be envisioned [58], and in turn what kind of health workforce is needed. Present and future funding levels are likely to be meagre, which weighs against the maintenance of a conventional, large public health service delivery structure. Investing available public resources in stewardship and regulation, while leaving service delivery to private providers, is conceptually appealing, but fraught with difficulties, as demonstrated by state administrations much stronger than the one under scrutiny.

*Using carrots where no sticks are available*. Subsidies might motivate key actors to change behaviour; unenforceable regulatory provisions will not, and may make matters worse. But effective incentives need an intimate knowledge of the market and prompt reactions to changing conditions. External financial assistance, already the source of the largest share of public resources, can in principle steer the healthcare arena in desirable directions, once the extent of privatisation and commoditisation is recognised and provided its inputs are used coherently in a long-term, firmly contextualised and strategic way. Aid could be used to motivate training institutions to raise quality standards against a reduction of enrolment numbers.

Affordability and workloads rather than international ratios (obviously beyond reach for a country as poor as Guinea-Bissau) should indicate the number of health workers to be trained. Hardship and productivity rewards would offset the progressive numerical slimming of the workforce*.* But better skills could not translate into better practice if the interplay of incentives remains negative, that is, if healthcare practice is conditioned by earning pressures. In principle, fewer pairs of competent hands could be paid better. Competitive salary levels could be introduced after severing health workers from the civil service. Turning healthcare structures into autonomous bodies would facilitate such a process.

Meanwhile, the registration of active health workers should be promoted, through tests awarding qualifications perceived as advantageous in the labour market, for instance by being preferred for recruitment by NGOs and charities. The resulting information would then be used for designing in-service and upgrading training programmes aimed at raising professional standards. Any HRH development plan should recognise the internationalisation of the health labour market and in turn the limits of domestic decision-making.

## Conclusions

Guinea-Bissau offers a telling example of how a national health workforce can deteriorate under protracted stress. Analysing health systems in undergoverned states is particularly testing, given the informalisation of key aspects of healthcare provision and the subsequent unreliability of official data. The present study aimed at analysing the HRH situation in Guinea-Bissau in light of the recent literature on distressed health systems, with the objective of contributing to identifying the forces at play, the resulting distortions and the counter-measures that might be considered. Through document analysis, focus group, semi-structured and in-depth interviews, we aimed at assessing how HRH react to protracted under-resourcing and mismanagement in the Guinea-Bissau settings.

Since independence, political turbulence has impacted on the evolution of the national health workforce, from the waves of diaspora following armed conflict and coup attempts, to subsequent mutually inconsistent rehabilitation programmes sponsored by aid agencies and their inevitable repercussions on the health market (Fig. 2). Chronic scarcity of funds and a ‘stable political instability’ emerged as two key forces shaping human resources for health in Guinea-Bissau for the last four decades. The commercialisation of public health services and flawed training and deploying mechanisms naturally ensued.

Solutions will always be hard to come by in situations similar to Guinea-Bissau’s; however, the hard-nosed recognition of its ingrained, if embarrassing distortions, coupled with an understanding of the incentives at play, will be essential starting points. Analysing in some detail the functioning of this de-regulated market will offer indications to set up incentives enticing health workers to perform better. Supervision and in-service training will identify the most serious skill gaps to be addressed in order to deliver better care. Stronger and timely information will enable quick adjustments. Investments in local management capacity might be needed to offset the paralysis of central health authorities (and of the central state administration), which might be beyond repair.

## Appendix 1

### Interview questionnaire (in Portuguese)

Bom dia,

Somos uma equipa do Instituto de Higiene e Medicina Tropical, e estamos a realizar um estudo sobre os RHS na Guiné. O estudo visa entender as razões da crise atual, com vista a identificar possíveis soluções.

**Ice-breaker**

Quais são as suas funções atualmente?

Qual é (ou foi) o seu contacto com a área dos recursos humanos?

Qual é a sua opinião sobre a situação atual em que se encontram os RHS no país?

Na sua opinião, quais serão os 3 constrangimentos principais da força de trabalho da saúde neste momento?

**Evolução histórica**

1. Na sua opinião a situação era melhor ou pior antes?
2. Pode identificar as que considera serem as etapas marcantes da evolução dos RHS ao longo das últimas décadas?
  i. Probe: Independência, Guerra, golpes de estado específicos etc.

**Distorções-chave**

**Composição da força de trabalho**

3. Na sua opinião, quando um doente procura serviços de saúde, para quem se dirigirá primeiro?
4. Quem são os prestadores de serviços de saúde fora das estruturas públicas?
  - Probe: Quem presta serviços de saúde nas zonas rurais e nos bairros?
5. Onde é que se poderão comprar medicamentos nas cidades e nas zonas rurais?
6. Que tipo de pessoal é que se encontrará no sector privado?
  - Probe: as qualificações e formação deles
7. E nos hospitais e centros de saúde público que tipo de profissional se encontrará?
8. Qual é a sua opinião sobre a qualidade dos profissionais que se encontram no público?
9. De que tipo de quadro é que se sente particularmente falta?
10. Porquê é que as vezes há pessoal formado que não se consegue colocar?

**Comercialização dos serviços de saúde**

11. Quanto é que o senhor/a acha que gasta em saúde por ano?
12. Quanto custa uma consulta/operação no público?
  - Probe: as cobranças ilegais e os medicamentos
13. Sai mais barato procurar no privado?
14. Quanto tipos de sector privado conhece?
  - Probe: áreas urbanas e rurais

**Fluxos de recursos e financiamento no sector saúde**

15. Onde é que as pessoas encontram o dinheiro para pagar os serviços de saúde?
  - Probe: remittances, emprestamos etc.
16. Os profissionais de saúde queixam-se que não recebem salários ao fim do mês; quem deveria paga-los?
17. E as ONGs e igrejas ajudam nisto? De que forma?

**Pressões externas**

18. Que doadores conhece específicos da área da saúde?
19. Conhece profissionais de saúde trabalhando para ONGs ou igrejas?
20. Que tipo de trabalho de saúde é que se faz nessas organizações?
21. Acha que as condições oferecidas aos trabalhadores são diferentes nessas organizações/igrejas?
22. No seu entender, porquê estas instituições atuam na área da saúde?

**Situação política e saúde**

23. De que forma acha que a situação política atual está a prejudicar os trabalhadores da saúde?
24. Os serviços estarão suspensos em algumas partes do país por causa da segurança?
25. O que acontece na zonas onde não há segurança aos serviços de saúde e aos trabalhadores?
26. Conhece algum plano de reforma da força de trabalho da saúde?
  - Probe: PNDRHS, porquê não foi implementado

## Appendix 2

### Work history interview guide

1. Tell me a bit about yourself? How did you come to work in the health field?
2. What kinds of jobs have you done in the past?
3. How did you get this job?
4. Where did you receive your training?
5. Where were you deployed after training? Probe: Did you accept the destination, or tried to have it changed?
6. How long have you been working here? *Probe for number of years or events that occurred when the participant started working in the facility.*
7. Given your position in this facility, please describe(briefly) what your roles and responsibilities are
8. Tell me about the different kinds of pay which you receive (probe: salary; allowances; user fees; payments from patients; incentives for deliveries; private business etc.).
  - Which ones are most valuable for you?
  - Why?
  - How do they change the way you work?
9. Apart from this job, do you have any other jobs/activities that you do/carry out as another source of income? Tell me about them
10. How do you feel about your current job? What do you like and dislike about it?
11. Are you planning to stay and work at this health facility? *If yes, probe for the reasons why. If no, probe for the reason why the participant may chose to move away from this work station.*
12. What plans do you have for your future career?

## Abbreviations

CHW: Community health worker
ENS: National Health School
GDP: Gross domestic product
HRH: Human resources for health
HW: Health workforce
MoH: Ministry of Health
MoPH: Ministry of Public Health
NGI: Non-governmental institution
NGO: Non-governmental organisation
PI: Political instability
PNDRH: National Human Resources Development Plan
PNDS: National Health Sector Development Plan
SAB: Bissau City Autonomous Area
USD-PPP: Purchasing Power Parity American Dollars

## References

[1] Dussault G, Dubois C-A (2003). Human resources for health policies: a critical component in health policies. Hum Resour Health.

[2] Chen L, Evans T, Anand S, Boufford JI, Brown H, Chowdhury M (2004). Human resources for health: overcoming the crisis. Lancet.

[3] Pavignani E. Beyond the aid horizon: charting poorly understood health territories. University of Queensland and DANIDA; 2014. (Findings from a multi-country research programme on health service delivery in severely-disrupted countries).

[4] Pavignani E, Colombo S (2009). Analysing disrupted health sectors. A modular manual.

[5] Fujita N, Zwi AB, Nagai M, Akashi H (2011). A comprehensive framework for human resources for health system development in fragile and post-conflict states. PLoS Med.

[6] WHO. Guide to health workforce development in post-conflict environments [Internet]. WHO Press, World Health Organization, 20 Avenue Appia, 1211 Geneva 27, Switzerland; 2005 [cited 2016 Apr 21]. Available from: http://www.who.int/hac/techguidance/tools/guide%20to%20health%20workforce%20development.pdf

[7] Roome E, Raven J, Martineau T (2014). Human resource management in post-conflict health systems: review of research and knowledge gaps. Confl Health.

[8] Witter S, Wurie H, Bertone MP. The free health care initiative: how has it affected health workers in Sierra Leone? Health Policy Plan. 2015;31(1):1–9. 10.1093/heapol/czv006 PMC4724164 25797469

[9] Bertone MP, Samai M, Edem-Hotah J, Witter S (2014). A window of opportunity for reform in post-conflict settings? The case of Human Resources for Health policies in Sierra Leone, 2002–2012. Confl Health.

[10] Bertone MP, Witter S (2015). An exploration of the political economy dynamics shaping health worker incentives in three districts in Sierra Leone. Soc Sci Med.

[11] Witter S, Falisse J-B, Bertone MP, Alonso-Garbayo A, Martins JS, Salehi AS, et al. State-building and human resources for health in fragile and conflict-affected states: exploring the linkages. Hum Resour Health [Internet]. 2015 May 15 [cited 2016 Jan 21];13. Available from: http://www.ncbi.nlm.nih.gov/pmc/articles/PMC4488955/10.1186/s12960-015-0023-5 PMC4488955 25971407

[12] Bertone MP, Lagarde M. Sources, determinants and utilization of health workers’ revenues: evidence from Sierra Leone. Health Policy Plan. 2016;31(8):1010–1019. 10.1093/heapol/czw031 PMC5013780 27053639

[13] Bertone MP, Lurton G, Mutombo PB. Investigating the remuneration of health workers in the DR Congo: implications for the health workforce and the health system in a fragile setting. Health Policy Plan. 2016;31(9):1143–1151. 10.1093/heapol/czv131 26758540

[14] Hill PS, Pavignani E, Michael M, Murru M, Beesley ME (2014). The “empty void” is a crowded space: health service provision at the margins of fragile and conflict affected states. Confl Health.

[15] Kok MO, Rodrigues A, Silva AP, de Haan S (2012). The emergence and current performance of a health research system: lessons from Guinea Bissau. Health Res Policy Syst BioMed Cent.

[16] Fistein D (2011). Guinea-Bissau: how a successful social revolution can become an obstacle to subsequent state-building. Int J Afr Hist Stud.

[17] Anderson L. Antiquated before they can ossify: states that fail before they form. J Int Aff [Internet]. 2004 [cited 2016 May 17];58(1). Available from: http://www.columbia.edu/itc/journalism/stille/Politics%20Fall%202007/Readings%20--%20Weeks%201-5/LEH57JE3248.pdf

[18] de Barros M, Gomes PG, Correia D (2013). Les conséquences du narcotrafic sur un État fragile : le cas de la Guinée-Bissau. Altern Sud.

[19] Abdenur AE, Neto DMDS (2014). Rising powers and the security-development nexus: Brazil’s engagement with Guinea-Bissau. J Peacebuilding Dev.

[20] The World Bank. Guinea Bissau country overview [Internet]. 2015 [cited 2016 Feb 17]. Available from: http://www.worldbank.org/en/country/guineabissau/overview

[21] UNDP. Human development report. New York: United Nations Development Program; 2013. Report No.: ISBN 978-92-1-126340-4.

[22] IMF. Guinea-Bissau IMF country report [Internet]. Washington, DC: International Monetary Fund; 2015 [cited 2016 Apr 27]. (Selected Issues). Available from: https://www.imf.org/external/pubs/ft/scr/2015/cr15195.pdf

[23] MINEF. Lei do Orçamento Geral do Estado 2015 [Internet]. Ministério de Economia e Finanças; 2015 [cited 2016 Apr 27]. Available from: http://www.mef-gb.com/doc/OGE/OGE2015.pdf

[24] MINISTÉRIO DA ECONOMIA E FINANÇAS, DIRECÇÃO GERAL DO PLANO INSTITUTO NACIONAL DE ESTATÍSTICA (INE). Inquérito aos Indicadores Múltiplos (MICS) 2014, Principais Resultados. Bissau; 2015

[25] Ministério da Saúde Pública da República da Guiné-Bissau. Plano Nacional de Desenvolvimento Sanitário I. 1997

[26] MINISTÉRIO DA SAÚDE PÚBLICA DA GUINÉ-BISSAU. Plano Nacional de Desenvolvimento Sanitário II 2008-2017. 2007

[27] MINISTÉRIO DA ECONOMIA, DO PLANO E DA INTEGRAÇÃO REGIONAL DA REPÚBLICA DA GUINÉ-BISSAU. Deuxième Document de Stratégie Nationale pour la Réduction de la Pauvreté-DENARP II 2011-2015. Bissau; 2011

[28] Ferrinho P. Subsídios para a revisão do Plano Nacional de Desenvolvimento Sanitário 2008-2017 até 2020. Lisbon; 2015

[29] Pavignani E, Michael M, Murru M, Beesley ME, Hill PS (2013). Making sense of apparent chaos: health-care provision in six country case studies. Int Rev Red Cross.

[30] Bloom G, Standing H, Lucas H, Bhuiya A, Oladepo O, Peters DH (2011). Making health markets work better for poor people: the case of informal providers. Health Policy Plan.

[31] Russo G, McPake B, Fronteira I, Ferrinho P (2014). Negotiating markets for health: an exploration of physicians’ engagement in dual practice in three African capital cities. Health Policy Plan.

[32] Beesley M, Cometto G, Pavignani E (2011). From drought to deluge: how information overload saturated absorption capacity in a disrupted health sector. Health Policy Plan.

[33] Pavignani E (2011). Human resources for health through conflict and recovery: lessons from African countries. Disasters.

[34] Tong A, Sainsbury P, Craig J (2007). Consolidated criteria for reporting qualitative research (COREQ): a 32-item checklist for interviews and focus groups. Int J Qual Health Care J Int Soc Qual Health Care ISQua.

[35] Harrell MC, Bradley MA. Data Collection Methods [Internet]. 2009 [cited 2016 Mar 29]. Available from: http://www.rand.org/pubs/technical_reports/TR718.html

[36] Wurie HR, Samai M, Witter S (2016). Retention of health workers in rural Sierra Leone: findings from life histories. Hum Resour Health.

[37] McKeown J, Clarke A, Repper J (2006). Life story work in health and social care: systematic literature review. J Adv Nurs.

[38] Coovadia H, Jewkes R, Barron P, Sanders D, McIntyre D (2009). The health and health system of South Africa: historical roots of current public health challenges. Lancet.

[39] Chabot J, Waddington C (1987). Primary health care is not cheap: a case study from Guinea Bissau. Int J Health Serv.

[40] The World Bank. Cost recovery in public health services in sub-Saharan Africa [Internet]. The World Bank; 1995 [cited 2016 Feb 17]. 114 p. (World Bank Institute Resources). Available from: http://elibrary.worldbank.org/doi/abs/10.1596/0-8213-3240-6

[41] MINSAP. Plano Nacional de Desenvolvimento Sanitário 1997-2001. Tomo 1 [Internet]. Ministério da Saúde Pública, Governo da Guiné Bissau; 1996 [cited 2016 Feb 22]. Available from: http://www.guine-bissau.fi/saude/5.2.html#_ftn30

[42] MINSA. Plano Nacional de Desenvolvimento Sanitário 2012-2025. Ministério da Saúde de Angola; 2014

[43] DNRH. Base de dados dos trabalhadores da função pública da saúde. Ministério da Saúde Pública, Governo da Guiné Bissau; 2016.

[44] Ammar W (2009). Health beyond politics.

[45] Meessen B, Bigdeli M, Chheng K, Decoster K, Ir P, Men C (2011). Composition of pluralistic health systems: how much can we learn from household surveys? An exploration in Cambodia. Health Policy Plan.

[46] Sen K, Mehio-Sibai A (2004). Transnational capital and confessional politics: the paradox of the health care system in Lebanon. Int J Health Serv Plan Adm Eval.

[47] Buckley J, Neill E, Aden AM. Assessment of the private health sector in Somaliland, Puntland and South Central. 2015 [cited 2016 Jul 4]; Available from: http://r4d.dfid.gov.uk/Output/202331/Default.aspx

[48] Indjai B, Catarino L, Mourão D. Mezinhos de Orango - Plantas medicinais e pessoas da ilha da Rainha Pampa. Bissau: Instituto da Biodiversidade e das Áreas Protegidas; 2010

[49] MINSAP. Plano Nacional de Desenvolvimento Sanitário 2008-2017 - PNDS II. Ministério da Saúde Pública, Governo da Guiné Bissau; 2008.

[50] Schaaf M, Freedman LP (2015). Unmasking the open secret of posting and transfer practices in the health sector. Health Policy Plan.

[51] ENS. Número de formados da ENS na Direcção de Dr. Maram Mané. Ministério da Saúde Pública, Governo da Guiné Bissau; 2016.

[52] Knippenberg R, Alihonou E, Soucat A, Oyegbite K, Calivis M, Hopwood I (1997). Implementation of the Bamako Initiative: strategies in Benin and Guinea. Int J Health Plann Manage.

[53] McPake B, Hanson K, Mills A (1993). Community financing of health care in Africa: an evaluation of the Bamako initiative. Soc Sci Med.

[54] Karadaghi G, Willott C. Doctors as the governing body of the Kurdish health system: exploring upward and downward accountability among physicians and its influence on the adoption of coping behaviours. Hum Resour Health [Internet]. 2015 Jun 4 [cited 2017 Jan 5];13. Available from: http://www.ncbi.nlm.nih.gov/pmc/articles/PMC4464857/10.1186/s12960-015-0039-x PMC4464857 26041465

[55] Tyrrell AK, Russo G, Dussault G, Ferrinho P (2010). Costing the scaling-up of human resources for health: lessons from Mozambique and Guinea Bissau. Hum Resour Health.

[56] Beesley M. The bottom of the sack: health service provision in the Central African Republic. DANIDA; 2013

[57] Pavignani E. Making sense of apparent chaos: health-care provision in six country case studies - ICRC [Internet]. International Review of the Red Cross. 00:00:00.0 [cited 2016 Apr 22]. Available from: https://www.icrc.org/eng/resources/documents/article/review-2013/irrc-889-pavignani-michael-murru-beesley-hill.htm

[58] Segall M (1991). Health sector planning led by management of recurrent expenditure: an agenda for action-research. Int J Health Plann Manage.

